# The Effectiveness of a Mindfulness Training Program on Selected Psychological Indices and Sports Performance of Sub-Elite Squash Athletes

**DOI:** 10.3389/fpsyg.2022.906729

**Published:** 2022-07-29

**Authors:** R. S. K. Wong, P. N. How, J. P. G. Cheong

**Affiliations:** ^1^MYWellness and Sport Science Consulting, Kuala Lumpur, Malaysia; ^2^National Sports Institute, Kuala Lumpur, Malaysia; ^3^Centre for Sport and Exercise Sciences, University of Malaya, Kuala Lumpur, Malaysia

**Keywords:** mindfulness acceptance commitment, single case research design, Malaysia, visual analysis, elite athletes

## Abstract

Mindfulness Acceptance Commitment (MAC) programs have garnered much support in enhancing sport performance through present-moment focus and non-judgmental thoughts. Expanding on previous studies conducted in collegiate and professional settings, the current study investigates the application of MAC amongst national sub-elite athletes. The study was conducted utilizing a single case A-B design, with a total of six sub-elite Malaysian Squash athletes (2 males, 3 females; M_age_ = 15 ± 2 years) purposively sampled from the Malaysian national squash team. Participants underwent 6 weeks of baseline testing, 7 weeks of program intervention, and a retention test 4 weeks post-intervention. The intervention consisted of psycho-education, centering and cognitive defusion among other aspects as purported in MAC programs. Changes in proficiency of mindful practice was observed through the Mindfulness Awareness Acceptance Scale (MAAS), experiential avoidance through the Acceptance Action Questionnaire (AAQ-II), stress levels through the Perceived Stress Scale (PSS), and sport performance through both coach- and self-rated scales. Overall, visual analysis revealed improvements in MAAS levels (M = 1.15 ± 0.15), with no marked changes in AAQ-II (M = –0.002 ± 1.12) and PSS (M = 0.7 ± 0.93) after 7 weeks of intervention. Coach-rated sport performance also improved across the phases (M = 0.86 ± 0.93), with mixed responses for self-rated improvements (M = 0.01 ± 1.19). Overall, the benefits of MAC program were well-maintained past the post-intervention phase. The current study supported the implementation of an MAC program for sub-elite athletes in real-world settings.

## Introduction

Mindfulness is a process often linked to the practice of mindful meditation (Kabat-Zinn, 1994). To be precise, mindfulness is a general construct described as paying attention in a particular way, “on purpose, in the present moment, and non-judgmentally,” whereas mindful meditation is the application of the aforementioned components in the practice of meditation (Kabat-Zinn, 1994). Mindfulness has been conceptualized as both a state (temporal) during the practice of mindful meditation (Lau et al., 2006), and a trait referring to an individual’s tendency to be mindful in daily life (Baer et al., 2006). Mindfulness has been posited to affect sport performance through bolstering of one’s psychological skills. In Birrer et al.’s (2012) review, they proposed 9 pathways in which mindfulness facets influence a variety of psychological skills, including bare attention, attitude, self-regulation and less rumination among others. Most studies concur on the two defining components of mindfulness: (1) paying attention to the present moment (bare attention), and awareness of the present moment experience, and (2) acceptance of the ever-changing moment-to-moment experiences (Bishop et al., 2004; Quaglia et al., 2015). This method differs from conventional psychological skills training (PST) approaches to stressful experiences. As an example, faced with a stressful competition, an athlete predisposed to using PST may tend to develop strategies that orient attentional resources toward controlling or purposefully reducing feelings of anxiety, whereas an athlete trained in mindfulness would simply accept the experience as it is and focus attentional resources on what is productive at the present moment. Studies examining neural responses to developing these two components of mindfulness (bare attention and acceptance) observe enhanced working memory and subsequently mental efficiency (Marks, 2008; Hölzel et al., 2011). Assuming these attentional resources being finite (Adam and Serences, 2019), it further enhances the athletes’ efficiency in allocating mental resources directed to competition stimuli without the need for excessive rumination (and redirection of mental resources) on negative internal states. The benefits from more efficient use of mental resources transfer over to optimal performance among elite athletes (Bertollo et al., 2016), further supporting the adoption of mindfulness and acceptance-based approaches in promoting sport performance.

As yet, mindfulness is thought to be trainable through structured and planned practice of mindful meditation (Visted et al., 2014; Creswell, 2017). The idea that mindfulness may serve better in optimizing cognitive resources and psychological states have driven sport psychology practitioners and performance coaches to reassess their prior approaches to working with the mental side of performance (Gardner and Moore, 2020). In fact, packages of mindfulness training programs have since emerged as the consequence of increased scientific interest in the application of mindfulness for improving health and performance. To date, mindfulness based programs featured in studies include Mindfulness Based Stress Reduction (MBSR; Kabat-Zinn, 1994), Mindful Sport Performance Enhancement (MSPE; Kaufman et al., 2009), and Mindfulness Acceptance Commitment (MAC; Gardner and Moore, 2004) among other more clinically-oriented approaches. Briefly, the MBSR is the pioneering program designed to cultivate mindfulness aimed at stress reduction, an approach emergent at the time of its inception. The MSPE expands on the MBSR with more contextual focus on enhancing sport performance, emphasizing development of mindfulness techniques and a sport-specific meditation that drives attention to sensations and motions an athlete may experience in their sport. Notably, the MSPE has mostly been utilized in research with target sports such as archery, golf and running which are mostly closed skill sports (Thompson et al., 2011). This is in tandem with the original contention of the proponents who intended MSPE for use with sports requiring sustained concentration and fine-motor skills (Wolanin and Gross, 2016).

Contrastingly, the Mindfulness Acceptance Commitment (MAC) program, which debuted in 2001 as an alternative for sport psychology interventions, had the objective of promoting positive and productive impact on sport performance while improving psychological health and well-being (Gardner and Moore, 2004). MAC draws on practices from both mindfulness training and Acceptance Commitment Therapy (ACT; Hayes et al., 2006), with a focus on values, value driven behavior and commitment. As a structured mindfulness training program, MAC works on the premise that improved ability to pay mindful attention and experiential openness allowed the athlete to maximize mental and emotional resources in committing to goal-driven actions and behavior (Gardner and Moore, 2020). Findings across studies in recent years have lent empirical support toward this premise. Case studies observed a marked enhancement in awareness, attention and subsequently competitive performance among elite athletes following the completion of a MAC training protocol (Gardner and Moore, 2004; Gooding and Gardner, 2009; Schwanhausser, 2009; Zhang et al., 2016; Koh et al., 2021). Additionally, in a randomized controlled trial (RCT) study comparing the effectiveness of MAC and PST on improving both athletic performance and mental well-being among collegiate athletes, it was reported that there were significant improvements in coach-rated performance, and significant reductions in self-reported substance abuse and symptoms of distress (Gross et al., 2016). More recently, Dehghani et al. (2018) expanded on the applications of MAC by investigating its use in team sports within the collegiate setting in Iran and found attenuated sport-competition anxiety and improved sports performance, suggesting that the benefits of MAC were carried over even where team dynamics were concerned.

The emergence of mindfulness based programs in enhancing sport performance provided an avenue to observe its effects across different levels of sport competition. Considering the MAC approach as a viable method to enhance sport performance at the collegiate and professional level, it was therefore only sensible, if not imperative, to observe with rigor its effects in a national elite setting where the stakes and commitment may differ for the receiving athletes. Other than the professional league, national elite athletes are mandated to represent their respective countries at the highest level of competitions, including international and major multi-sport events such as the Asian, Commonwealth and Olympic Games. As such, compared to collegiate and professional sports (where phases of competitions may be more clearly segmented), national elite athletes are expected to perform and peak at perhaps more frequent intervals in an annual (note: not seasonal) program. National elite athletes carry the responsibility of not only personal performance, but also including national reputation. With only one other named study applying mindfulness practice with national level athletes (Mardon et al., 2016), more investigation is needed to ascertain the efficacy of mindfulness with this elite population. Within the context of the current study investigating squash athletes, it was also notable that squash is dynamic and open-skill compared to most target sports, with more direct elements of confrontation between competitors in the court (Abernethy, 1990; Jones et al., 2018). With sport performance being dynamic and a stressful competitive training environment, three consequential variables were deemed important for the current study: (1) proficiency in mindfulness, (2) experiential avoidance, (3) perceived stress levels, and (4) sport performance. Proficiency in mindfulness represents the ability and efficacy of the athlete to utilize mindfulness for psychological gain, experiential avoidance as the desire to control or suppress unwanted experiences, perceived stress as representation of overall stress from training and competition, and sport performance as the object of improvement for the athletes. As such, the MAC approach was deemed more viable for the purposes of this study. The current study provides a reference point for national level coaches working with elite and specifically sub-elite athletes in consideration of onboarding a mental performance program with a week-by-week approach to manage expectations. Concurrently, the current study presents a spectrum of findings across 6 participants with varying effects yielded from the MAC program, explored at the individual level. Furthermore, specific published studies examining its application in competitive racquet sports setting, including squash, remain sparse.

The current study observed the application of MAC in a group of sub-elite squash athletes in an applied national level setting, with the objective of observing its effects on the athletes’ sport performance and well-being. For brevity, the study aimed to determine the effects of an MAC intervention program on selected psychological variables (i.e., proficiency in mindfulness, experiential avoidance and psychological inflexibility, perceived stress) and sports performance (self- and coach-rated). Visual analysis (VA) was employed for its strength in elucidating changes in a single case-design with a week-to-week breakdown, which is particularly suited for the current study’s high level population.

## Materials and Methods

### Research Design

The study was a quasi-experimental research utilizing a single subject, A-B design involving a Mindfulness-Acceptance-Commitment (MAC) intervention. A single-case design, with an A-B design across-participants (Barker et al., 2011) was adopted as it allowed the intervention to be compared to stable baseline data for each participant individually prior to the intervention. In this design, participants underwent an MAC intervention program for a duration of 7 weeks. Retention of effects was measured 4 weeks post-intervention, by comparing the value of each measurement during post-intervention to the peak value in the baseline phase.

### Participants

Participants were volunteers from a pool of Malaysian sub-elite squash athletes. To be included in the study, the participants had to be injury and concussion-free, actively training and competing in the Professional Squash Association, and had no prior exposure to mindfulness based modalities. It was deemed necessary for participants to complete all sessions and measurements, failure of which resulted in exclusion from the study. Whilst a total of 10 athletes volunteered to participate in the study, only six (two males and four females) completed all phases of the research and were included in this study. These internationally ranked sub-elite athletes live and train in a regimented lifestyle similar to their elite counterparts competing in national and international level competitions. The participants have represented Malaysia at major games such as South East Asia Games, Commonwealth Games and World level age-group championships age ranging between 13 and 17 years old). All had undergone formal learning of PST with a Sport Psychologist for at least 5 years. Table 1 presents an overview of the participants’ demographics and level of performance in competitions. The parents/guardians of the participants signed the informed consent form and the study was carried out according to University ethical guidelines (Ethical approval reference number UM.TNC2/UMREC-161).

**TABLE 1:** Participants demographic information and championships results.

Athlete	Participation competition	Results	Gender/Age
1	British Junior Squash Championships Age group National Circuit	Medalist Medallist	Female/16
2	British Junior Squash Championships Age group National Circuit	Medalist Medallist	Female/16
3	British Junior Squash Championships Age group World Junior Championships South East Asia Games National Circuit	TOP 8 Gold Medalist Medalist	Female/16
4	British Junior Squash Championships Age group World Junior Championships South East Asia Games National Circuit	TOP 8 Gold Medalist Medalist	Female/15
5	British Junior Squash Championships Age group World Junior Championship South East Asia Games National Circuit	TOP 8 Gold Medalist Medalist	Male/17
6	British Junior Squash Championships Age group National Circuit	TOP 8 Medalist	Male/13

### Data Collection

Measures comprised of psychological indices of mindfulness, well-being and sport performance. Data collection spanned a total duration of 17 weeks (6 weeks for baseline, 7 weeks for intervention and 4 weeks for post-intervention to measure retention effects). Notably, an interstate competition took place during week two of the intervention phase, and as such, one data point of the intervention data was collected during the competition. To measure retention of effects, the value for each measurement at post-intervention phase was compared with the peak value in the baseline phase (with the exception of Acceptance Action Questionnaire (AAQ-II) and Perceived Stress Scale (PSS) as these were negatively scored, ie. lower AAQ-II scores signified lower experiential avoidance, which was desirable in the current study).

### Measures

Five measures were taken in total: one for proficiency in mindful practice, one for experiential avoidance, one for perceived stress, and two for sport performance. For standardization, all measures were presented and taken in English.

#### Sports Performance

##### Coach’s Rating Scale of Athlete’s Performance

One squash coach provided performance ratings of the participants using a scale abbreviated from Wolanin’s (2004) MAC study. The rating scale consisted of 11-items and used a 7-point Likert scale that ranged from (1) *very poor* to (7) *extremely good*. The rating scale was a direct measure of athletic performance and consisted of the following performance variables: concentration, strength, competitiveness, motivation, quickness, fitness, endurance, mechanics, aggressiveness, agility, and team cohesion. Ratings were carried out at the end of each week where an aggregated score from all 10 items were taken into measurement as the final performance score for the athlete. Examples of variable descriptions were agility, graceful, nimble, flexible movement, ease of movement.

##### Athlete’s Performance Self-Rating Scale

Each participant completed a self-rating scale of his or her athletic performance which was similar to the questionnaire for coach-rated performance, with the only difference being the heading which was specific for the athletes. Participants completed the questionnaire at the end of each week. Examples of variable descriptions were mechanics, execution of skills and techniques necessary to perform the sport.

#### Psychological Indices of Mindfulness and Well-Being

##### Mindfulness Attention Awareness Scale (MAAS)

The questionnaire consisted of 15-items designed to assess trait mindfulness, specifically the tendency to attend to and be aware of internalized and environmental experiences in life (e.g., “I find myself preoccupied with the future or the past”) (Brown and Ryan, 2003). Responses were coded according to a 6-point Likert scale from (1) *almost always* to (6) *almost never.* Scores were totaled with higher scores reflective of the athletes’ tendency and proficiency in mindfulness practice. Internal consistency level was good, α = 0.87) and reliability was 0.81 (Brown and Ryan, 2003; Carlson and Brown, 2005). The questionnaire took 5–10 min to complete.

##### The Acceptance and Action Questionnaire-II (AAQ-II))

The AAQ-II consisted of 7-items used to measure experiential avoidance and psychological inflexibility (e.g., Emotions cause problems in my life). It was rated on a 7 -point Likert scale from (1) *never true* to (7) *always true*, with low scores indicating a low level of experience avoidance and psychological inflexibility, and vs. Validity of this measure was between 0.78–0.88, with good internal consistency (α = 0.88) and the 3- and 12-month reliability was 0.81 and 0.79, respectively (Bond et al., 2011).

##### The Perceived Stress Scale (PSS)

This 10-item questionnaire measured global stress based on the subject’s perception of stress (Cohen et al., 1983) in the past week (e.g., In the last week, how often have you felt that things were going your way?). It was measured on a 7-point Likert-type scale from (0) *never* to (6) *very often*. It is one of the most widely used psychological instrument for measuring stress that is general in nature rather than event-specific and takes approximately 5 min to complete. Internal consistency was acceptable, α = 0.74 (Lee, 2012). The scale also measured participant’s current levels of stress experiences.

### Mindfulness Intervention and Procedures

The MAC approach (Gardner and Moore, 2007) included a step-by-step protocol for the performance enhancement program. MAC training was a flexible approach consisting of seven sessions carried out in group sessions for this study. A general overview of session modules are shown in Table 2.

**TABLE 2:** Mindfulness Acceptance Commitment (MAC) 7-module content and learning outcomes done over 7 weeks of training.

No.	Sub-modules	Activities	Learning outcomes
1	Preparing the athletes with psychoeducation	Introduction to MAC The rationales of MAC The components of MAC The goals of MAC in performance enhancement Brief Centering (technique)	Identify the history of MAC development List the rationales of MAC approach Identify components of MAC approach Identified the characteristics of performance enhancement List the goals of MAC in performance enhancement
2	Introduction mindfulness and cognitive defusion	Rationale and importance of Mindfulness Cognitive fusion and cognitive defusion in mindfulness skills Cognitive defusion in performance enhancement Brief Centering (exercise) Mindful Breathing (exercise)	Discuss the level of self-and the rationales of mindfulness State the definition of mindfulness, cognitive, fusion and cognitive defusion Explain the role of Acceptance-Commitment Therapy(ACT) to improve mindfulness Identify the characteristics of cognitive fusion and cognitive defusion performance enhancement
3	Introducing values and values-driven behavior	Value situation and value driven behavior Brief centering exercise Value situation and value driven behavior for performance enhancement Brief centering exercised and mindfulness 1 Brief Centering (exercise) Mindful Breathing (exercise)	Explain the importance of value for determination commitment to a significant life situation Explain the unction of brief centering exercise in improving mindfulness Explain the purpose of value in mindfulness training Performing a brief centering and mindfulness technique Determine the value in performance enhancement
4	Introducing acceptance	Experiential acceptance Experiential acceptance in performance enhancement Brief centering exercise and mindfulness 2	Identify acceptance verbs in the process of cognitive defusion Comparing the difference in experiential acceptance and experiential avoidance Improve the level of brief centering and mindfulness Identify acceptance verbs in performance situations
5	Enhancing commitment	Connecting Values, Goal and Behavior Increase commitment to performance enhancement Brief centering exercise and mindfulness in performance	Identify which value and goal can change behavior Reuse the concept of cognitive fusion, cognitive defusion, experiential acceptance and value in producing a commitment to performance enhancement Improve brief centering exercise and mindfulness in performance
6	Skill consolidation and poise-combining mindfulness, acceptance and commitment	Brief centering exercise and mindfulness in performance Planning MAC strategy based on prediction situation on performance different scenarios	Designing appropriate MAC strategies based on performance scenarios Increase brief centering exercise and mindfulness in performance enhancement
7	Maintaining and enhancing mindfulness, acceptance and commitment	Brief centering exercise and mindfulness in performance Plan for future practice strategies	Mindfulness self-reflection and self-correction for performance enhancement Strategies for MAC approach practice and implementation for performance enhancement

The study was carried out in three phases. During baseline phase, participants completed five self-report questionnaires (MAAS, AAQ-II, PSS, Coach- and Self-rated performance) every Friday for 6 weeks through an online survey platform, whereas Coach-rated performance was obtained from the coach. The MAC program was then introduced in the following week on Friday, which counted as the start of the intervention phase. The intervention took place in a meeting room at the squash training center and took approximately 50 min. Sessions were held once a week for 7 weeks, carried out by one of the authors with over 10 years of experience working with elite-level racquet athletes.

The MAC program consisted of 7 modules, namely: (1) Preparing the athletes with psychoeducation: introducing the rationales, components and goals for the MAC program, (2) Introducing mindfulness and cognitive defusion: defining both components of the program and explaining its application in a sport context, (3) Introducing values and value-driven behavior: explaining the relation between goals, values and behavior, and differences between goal-oriented versus emotion-oriented behavior, (4) Introducing acceptance: explaining the implications of experiential avoidance in sporting context while exploring potential benefits to performance associated with being open to new experiences, (5) Enhancing commitment: explaining core differences between goals, motivation and commitment, and identifying key behaviors associated to improving performance, (6) Skill consolidation and poise: combining mindfulness, acceptance and commitment as a strategy for participants’ to practice on with performance-related scenarios, (7) Maintaining and enhancing mindfulness, acceptance and commitment: open discussion with the participants on maintaining their practice and challenges they came across for deeper learning.

In every session, mindfulness techniques were taught and practiced (e.g., brief centering, mindful breathing). With every mindfulness technique imparted, participants were encouraged to self-practice mindfulness techniques during off-training hours as homework at their own accord. Participants reported completion of self-practice through weekly check-in by text messaging, although no strict requirements were placed on them to do so. Measurements were maintained throughout the intervention phase taken consistently at the end of the training week (on Saturday). After 4 weeks of post-intervention phase, retention measurements were taken.

### Data Analysis

Visual Analysis (VA) was used to analyze the data of this study. VA involved visual examination of data for each participant to subjectively evaluate the reliable change in the outcome variable that can be observed (Jenny et al., 2014). Data obtained for each measurement were plotted on a line graph, where the horizontal (x) axis represented time (week) while the vertical (y) axis represented score for each outcome variable. By using the split middle method (White, 1974) as proposed by Kazdin (1982), trend lines were then drawn by “free-hand” method as a way of graphically illustrating changes within the graph.

Analysis was conducted on two forefronts: (1) within-condition analysis (i.e., Baseline phase, intervention phase), and (2) between-condition analysis which analyzes for changes between baseline and intervention phases. As the current study aimed to observe MAC effects upon onset (one week into intervention phase, hereafter referred to as “acute”) and overall effects between phases (hereafter referred to as “chronic”), comparison of between-conditions effect will be used to illustrate the intervention effect. In accordance with VA recommendations, analyzes were done to observe for changes in level, slope and trend, as well as stability and overlap (Hrycaiko and Martin, 1996).

#### Level

Level refers to the absolute value in a particular data point or phase of the outcome variables (i.e., MAAS, AAQ-II, PSS, performance) (Wolery and Harris, 1982; Lane and Gast, 2014). Acute changes were calculated by determining the ratio change between the final value of baseline phase (week 6 of baseline) from the first value of the intervention phase (week 1 of intervention) (Marlow et al., 1998). Between the two values, level change was calculated by dividing the largest trial score by the smallest, providing a description of the immediacy of MAC effects on outcome variables. For example, if a subject’s score for MAAS during the last point of baseline phase was 30, and 40 for first session of intervention phase, a ratio of x1.33 was observed, with “x” signifying a positive increase in slope upon intervention onset, and vice versa. Chronic changes were similarly calculated as ratio change, taking the overall median value of baseline and intervention phase. Analyzes for changes in level was reinforced by calculation of effect size using Cohen’s *d* following these guidelines: 0 to 20 = small, 20 to 50 = medium, more than 50 = large.

#### Slope and Trend

Slope refers to the rate of change within a phase, while trend refers to the general direction of the data pattern, which can occur in accelerating (upward, represented using the symbol “x”), decelerating (downward, represented by the symbol “÷”), or stable (little to no changes in direction) (Wolery and Harris, 1982; Lane and Gast, 2014). Both values are obtained from the celeration line through split middle analysis, by calculating the ratio change between median values of 1st half and 2nd half of each phase. Effect of intervention was determined by comparing the ratio change between baseline and intervention. For example, in baseline phase, a subject may have median values of 20 and 25 in 1st and 2nd half, respectively, which signifies a ratio change of x1.25 for baseline phase. As for intervention phase, the subject’s median values were 30 and 40, respectively, with a ratio change of x1.33. As the contrast of both celeration lines, the change in slope is therefore x1.064 (1.33÷1.25). This subject would then be summarized as having accelerating trend for both baseline and intervention phase, with an upward increase of x1.064 in slope at intervention phase, suggesting a stronger effect of intervention. It is entirely plausible for deceleration to occur in either phases. Ideally, intervention phase should exhibit an accelerating trend in MAAS, AAQ, and performance compared to baseline.

#### Stability

Stability alludes to how similar, or variable the scores are in a condition (Wolery and Harris, 1982; Morgan and Morgan, 2009; Lane and Gast, 2014). Stability of effects would signify reliability of treatment effects, and vice versa. Briefly, stability is determined when approximately 80% of values fall within 15 to 25% of the median value in a phase (this range is sometimes referred to as “stability envelope”) (Lane and Gast, 2014). The current study adopted a strict 25% stability envelope to ensure credibility of effects. Data overlap were also considered as measures of intervention validity, typically requiring 80% of data set in intervention phase to be: (1) above baseline levels (which were identified to be the highest value in baseline level for MAAS, Self-rated performance and Coach-rated performance) and (2) below baseline levels (lowest baseline value for AAQ-II and PSS) in order to determine intervention effect (Scruggs et al., 1987; Lane and Gast, 2014). As such, six points of data in the intervention phase were required to be above baseline levels to support the intervention effect for this study (i.e., 6/7 amounts to 85.71%).

## Results

### Psychological Indices of Mindfulness

**FIGURE 1:**
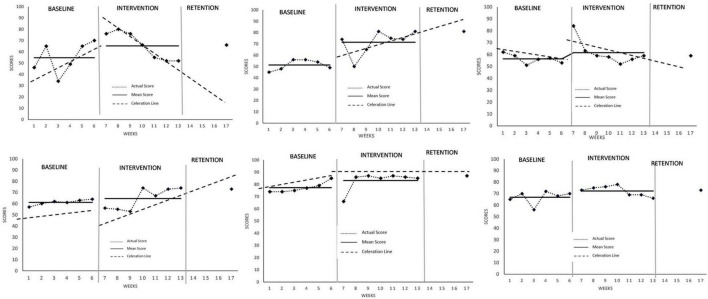
Mindfulness attention awareness scale (MAAS) scores across all participants.

Data from all six participants were analyzed to observe for differences, with all data deemed stable. Collectively, mean scores of trait mindfulness for all participants were observably higher across the intervention phase compared to baseline (Figure 1), signifying positive chronic changes for all participants in proficiency in mindfulness. Additionally, Cohen’s *d* effect size calculations for all participants were 0.79, 2.59, 0.73, 0.56, 1.00, and 1.07, respectively, pointing to the MAC intervention having a strong effect overall except for Athlete 4 (*d* = 0.56) who experienced a medium-strength effect. Changes in level occurred immediately for all participants, with only Athlete 3 and 4 experiencing a negative acute change at the rate of ÷1.14 and ÷1.29, respectively that rebounded onward of session 2 of intervention. Interestingly, only Athletes 2 and 4 experienced a positive change in slope and trend at x1.02 and x1.27, respectively, which implies a slowing down of positive changes for the other participants at varying degrees. Post-intervention data showed higher mean scores compared to baseline for all participants, signifying retention of positive effects on trait mindfulness.

### Experiential Avoidance and Psychological Inflexibility

**FIGURE 2:**
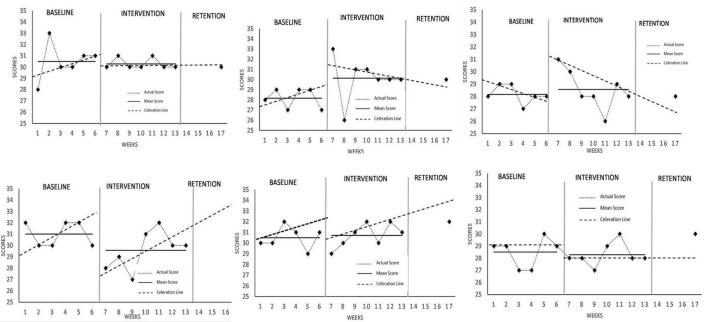
Acceptance and action questionnaire II (AAQ-II) scores across all participants.

Data set across all six participants were deemed stable. There were mixed findings overall for measures of experiential avoidance (Figure 2), with mean scores marginally lower across the intervention phase for Athletes 1, 4, and 6; marginally higher for Athletes 2 and 5, and stagnant for Athlete 3. Additionally, Cohen’s *d* effect size calculations for all participants were –0.2, 1.27, 0.34, –1.01, 0.19, and –0.19 for all participants, respectively, implying that the MAC intervention only had stronger effects for Athlete 2 and 4. Changes in level occurred immediately for all participants, with notably Athlete 2 and 3 experiencing an increase in experiential avoidance at the rate of x1.22 and x1.11, respectively. Only Athlete 1, 2, and 3 experienced a negative change in slope and trend at the rate of ÷1.03, ÷1.06, and ÷1.04, respectively, with the other 3 remaining stagnant. Post-intervention data showed scores that returned to baseline for all athletes except Athlete 1. Considering overall findings, the intervention had trivial effects on experiential avoidance.

### Perceived Stress Scale

**FIGURE 3:**
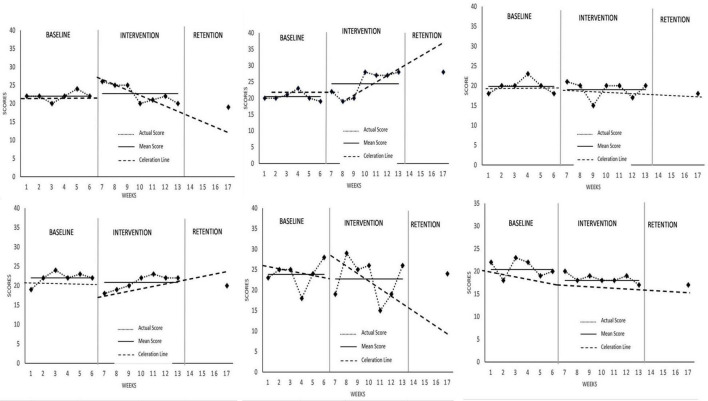
Perceived stress scale (PSS) scores across all participants.

Data set across all six participants were deemed stable. No changes were observed for all participants in perceived stress levels (Figure 3) in the intervention phase except for Athlete 2 who experienced an increase in PSS scores. This implied that the MAC intervention had otherwise no effect on mean perceived stress levels, with Athlete 2 presenting an isolated case. Cohen’s *d* effect size calculations for all participants were 0.37, 1.47, –0.42, –0.65, –0.27, and –1.63, respectively, signifying a strong effect for Athlete 2 and 6 (if any) but otherwise low to medium for the other participants. Changes in perceived stress level occurred immediately for Athlete 2 at the rate of x1.16. No acute changes were observed for Athlete 6. Notably, only Athlete 1 and 5 experienced a negative change in slope and trend at the rate of ÷1.19 and ÷1.28, respectively. Post-intervention data showed an increase in perceived stress level for Athlete 2, but otherwise no changes from baseline for the others. All data considered, MAC did not positively affect perceived stress levels, with Athlete 2 presenting a peculiar increase.

### Sports Performance

**FIGURE 4:**
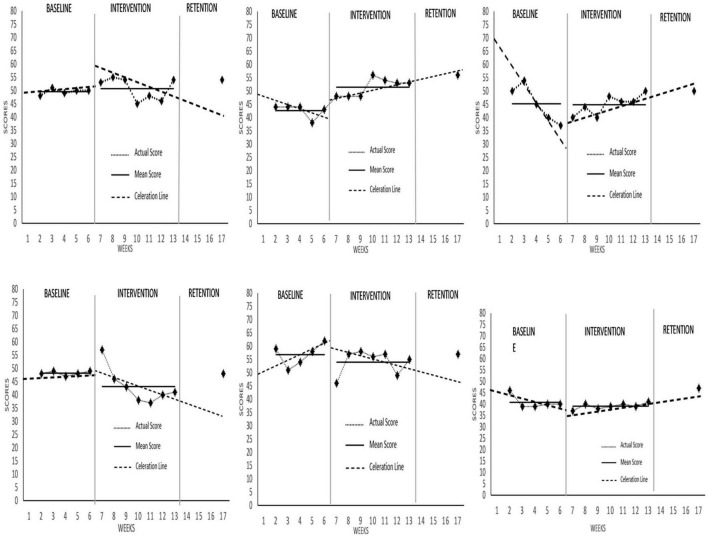
Self-rated performance scores across all participants.

Visual Analysis (VA) showed observable changes in self-rated performance in Figure 4, with all data deemed stable. Self-rated performance levels increased for Athlete 1, 2, and 3 and reduced for the Athlete 4, 5, and 6. Cohen’s *d* effect size calculations for all participants were 0.41, 2.96, –0.06, –1.32, –0.63, and –0.77, respectively, signifying strong positive effects for Athlete 2, strong negative effects for Athlete 4 and 6, and otherwise no effect for Athlete 5. Athlete 1, 2, 3, and 4 reported having acute increases with Athlete 5 and 6 having decreases at the rate of ×1.06, ×1.22, ×1.08, ×1.16, ÷1.35, and ÷1.08, respectively. Changes in slope and trend were positive for Athlete 2, 3 and 6 at the rate of ×1.11, ×1.40, and ×1.09, respectively Post-intervention data showed mixed findings, with Athlete 1, 2, and 6 having positive increases while the others returned to baseline. Overall, MAC had a mixed effect on self-rated performance, with Athlete 2 having the most increases compared to the others.

**FIGURE 5:**
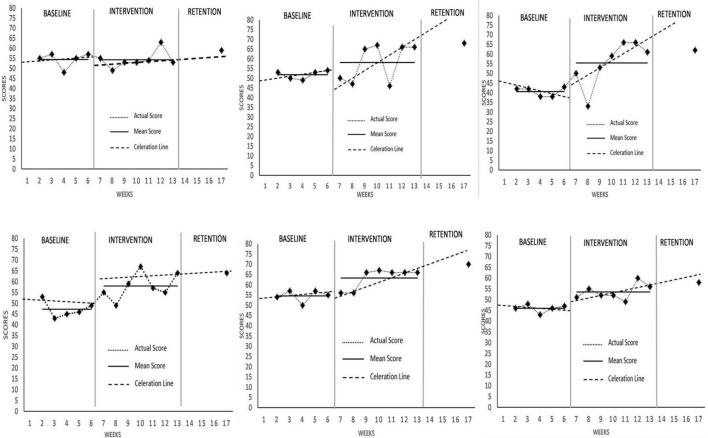
Coach-rated performance scores across all participants.

Comparatively, visual analysis showed observable changes in coach-rated performance in Figure 5, with most data deemed stable (except for Athlete 2’s intervention phase). All participants experienced an increased level of coach-rated performance except for Athlete 1. Effect size calculations (Cohen’s *d*) for all participants were –0.03, 1.05, 2.12, 2.18, 2.21, and 2.72, respectively, implying strong positive effects on coach-rated performance for all participants except Athlete 1. Changes in coach-rated performance level occurred immediately for all participants, with only Athlete 1 and 2 experiencing an acute decrease at the rate of –1.04 and –1.08, respectively that otherwise rebounded onward of session 2 for Athlete 2. Interestingly, changes in slope and trend were positive for all participants at the rate of×1.02,×1.28,×1.28,×1.05,×1.16, and×1.09, respectively. Post-intervention data showed positive increases for all athletes. Collectively, MAC had a positive effect on coach-rated performance for all participants except for Athlete 1.

## Discussion

The current study aimed to investigate the effects of MAC training on sub-elite athletes’ level of proficiency in mindfulness practice (MAAS), experiential avoidance and psychological inflexibility (AAQ), perceived stress levels (PSS) and subsequent sport performance (self- and coach-rated) over time. Results show that, generally, MAC had a positive effect on athletes’ proficiency in mindful practice. This lends support to an already growing notion that MAC program improves trait mindfulness, a key characteristic defining of proficiency in mindful practice (Hasker, 2010; Josefsson et al., 2019). While Josefsson et al.’s (2019) RCT study established that emotion regulation mediatedthe relationship between mindfulness and athletic performance, the current study took an in-depth approach to observing mindfulness and sport performance alongside experiential avoidance and perceived stress. As MAC works on the premise that being able to direct attention efficiently facilitates athletic performance, it may also have inculcated in athletes the ability to be “economical” in allocating cognitive resources because there was no need to consciously avoid or mitigate negative stimuli (Gardner and Moore, 2012). This would in turn translate to greater available cognitive resources for sport-specific tasks, which is paramount for peak performance. The results also lend credence that trait mindfulness is malleable with a 7 weeks mindfulness program, albeit at different rates across each participant. Similar to most psychological traits, trait mindfulness has been posited to be stable in nature. However, trait mindfulness has been shown to increase with individuals practicing mindfulness who were on an upward trajectory in state mindfulness over a period of 8 weeks (Kiken et al., 2015). Through sufficient volume of intentional practice, it was speculated that evoking the state of mindfulness repeatedly throughout meditations increase the propensity toward being mindful in everyday life (Garland et al., 2010; Kiken et al., 2015). Even though the current study did not account for state mindfulness in its measures, it is speculated that the benefits from the MAC program evoked sufficient changes in state mindfulness for the participants to have positive changes in their trait mindfulness.

As observed, all six athletes in the present study reported surpluses in MAAS level with varying magnitudes for acute and chronic improvements. As measured in MAAS, all athletes reported improvements on their ability to direct attention in a constructive way that focuses on actions and decisions within their control, although the rate at which this occurred differed across the athletes, with three athletes displaying an upward trend during the intervention phase while the other three showed a downward trend. These changes were observed to be stable across all athletes in the intervention phase. This is consistent with other studies that similarly investigated benefits of mindfulness intervention programs in a sport setting, Hasker (2010), Mistretta et al. (2017), Josefsson et al. (2019), Zadeh et al. (2019).

Interestingly, Athletes 4 and 5 experienced an acute reduction in MAAS level immediately upon intervention onset. For Athlete 4, the drop persisted for 3 weeks to the level of baseline but immediately rebounded in the fourth week and was on uptrend beyond that. When considering the efficacy of the MAC program, the content and delivery’s appeal is subject to individual preferences, with weeks 1 and 2 being more theoretical in content while week 3 introduced a practical emphasis on “centering” and “mindful breathing.” In follow up discussion with Athlete 4, it was found that the theoretical aspects in weeks 1 and 2 were familiar but intangible, citing the practical aspects in week 3 as a catalyst for practicing mindful attention in a “relatable way,” which may explain the temporary reduction at intervention onset. As for Athlete 5, the acute reduction in MAAS level on week 1 of intervention was immediately attenuated by improvements in week 2 onward, with the athlete citing a “sudden personal event” that presented some emotional challenges, but rebounded beyond baseline level upon resolution of the event. Importantly, these two examples represent challenges in the delivery of not only MAC, but possibly for other psychological interventions as well, where content and context plays a crucial role in mediating learners’ progress (Taie, 2014). Even if learning progress seems to slow down for a certain few, accounting for individual differences may yield beneficial gains later in the timeline, or even beyond. Athletes 4 and 5 were also two of the only three athletes displaying a positive change in trend despite an initial drop in level during intervention onset. Upon successful completion of MAC, it was observed that all athletes’ improvement were maintained after termination of the MAC program, with Athlete 6 experiencing additional gains during this period. This post-intervention improvement was similarly observed in Zadeh et al.’s (2019) study where improvements in mindfulness proficiency among semi-pro soccer athletes actually sustained upwards of 3-months. In fact, injury rates were observed to reduce following MAC program in Zadeh et al.’s (2019) study, followed by an improvement in athletic performance up to three months after MAC program, positing that proficiency in mindfulness may have had positive effects on athletes’ robustness toward injury. In a similar vein, Naderi et al.’s (2020) study found similar results with significantly lower injury rates for athletes in the MAC group, albeit across a competitive season. This was attributed to better mindfulness, attentional control and lower sport anxiety, which was in tandem with the current study’s variables of interest. In the current study, MAAS retention, which took place during a non-competition phase of training cycle, was measured 4-weeks post MAC program. This could reflect benefits yielded which were maintained through the athletes’ ability to be independent and self-organize their mindfulness practice even without supervision.

Conversely, the added responsibility of actively practicing mindfulness during the intervention period may have been perceived as a “hassle,” which may have confounded the observations during the initial phase of intervention. By way of the environment and past experiences, it is plausible that an athlete may have been rooted in depending on negative emotions or experiences to drive attention, and may present with confounding level of proficiency in directing attention during the baseline. Notably, as part of the inclusion criteria, all athletes have not been exposed to mindfulness concepts and practices before. Therein lies two risks: (1) the athlete may have a different understanding of “directing attention” before the start of the MAC program and (2) an initial decline in the skill or performance may be necessary in order for old habits and preferences to be replaced. Future studies can consider more elaborate designs to ascertain the intervention effect. An A-B-A-B design would allow more precise comparisons between periods of exposure to the MAC program, although this may only be possible in lab settings where improving sport performance is not the main objective. In high performance environments, future studies can consider a pre-test screening for attentional styles to further ascertain subjects’ innate characteristics. The Test of Attentional and Interpersonal Styles (TAIS; Nideffer, 1976) allows insight into a subject’s attentional preferences, which has been shown to have consequences for cognitive strategies during performance (Baghurst et al., 2004).

The current study projected improvements in experiential avoidance through reductions in AAQ scores. However, as observed in results, chronic changes (from phase to phase) in level between baseline and intervention phases were negligible across all subjects if not accounting for trend direction. In fact, changes in trend were curiously neutral compared to the projected effects of MAC program, with three athletes displaying a decelerating trend during intervention phase while the remaining three athletes displaying no changes at all. Ideally, reductions in AAQ level would signify an internalization of mindfulness practice into the athletes’ world-view and values, where they would be more actively engaged in goal-directed behavior rather than avoiding uncomfortable outcomes (such as “trying hard to avoid feeling depressed or anxious” or “needing to have doubts worked out in order to be productive”) (Bond et al., 2011). However, this was not the case in this study, and could be due to an already low initial baseline level of experiential avoidance in a scale that ranges between 16 (being lowest) to 112 (highest), hence further reductions could not be seen in an overt manner. Assuming experiential avoidance was a function of the psychological trait of openness (Hayes et al., 2004; Giluk, 2009), coupled with established stability of AAQ-II levels across all athletes during the intervention phase, it is understandable that personality trait changes occurred at a much more gradual rate (Bucher et al., 2019) compared to skill acquisition, which is MAAS level in the context of the current study. A meta-analysis investigating the relationship between psychological traits and mental health treatment outcomes suggested that changes in traits occur over longer periods of treatment between one to five months, and differ across life developmental stages (Bucher et al., 2019). Comparatively, the MAC program typically occurs over 5 to 7 weeks (Gardner and Moore, 2007), suggesting that effects of MAC programs on mitigating experiential avoidance may take longer than the typical seven week period to be obvious. Furthermore, the findings of this study is also in contrast to other previous studies that found a decrease in experiential avoidance. For example, in a dissertation where participants were exposed to either MAC or PST treatment across 7 weeks, it was reported that the participants experienced lesser experiential avoidance compared to baseline (Hasker, 2010). Considering the current study’s subjects’ age, skill acquisition of mindfulness practice may have resonated more with subjects’ motivation to improve performance at a rapid rate rather than trying to change intrinsic personality traits (Schwanhausser, 2009; Greenberg and Harris, 2012).

In terms of perceived stress (PSS), it was expected that participants would have reduced levels following the MAC program. Overall, changes across the sample were negligible for acute to chronic, meaning that reductions in stress were perhaps unnoticeable, with the exception of Athlete 2. Once again, this could be due to already low-rated perceived stress levels during baseline for all participants. However, considering that the intervention was conducted during a less important competition, perhaps increased proficiency in mindful practice may have served to attenuate comparably lower stress levels, compared to common spikes in competitive anxiety before competitions (Mellalieu et al., 2006). This would signify an improved ability to tolerate perceived stress levels during stressful events, although it would be interesting to observe effects in major competitions of the training cycle. Stress levels were shown to reduce for 5 athletes to an even lower level than baseline, suggesting that the MAC program may have served to facilitate athletes’ self-regulation which sustained for at least 4 weeks post intervention. As for Athlete 2, stress levels were observed to be higher than baseline and intervention as well as post-intervention which could be due to increased internalized pressure to perform. Notably, Athlete 2 is the only one to display variability in PSS levels during the intervention phase, whereas the others were stable throughout. Typically, this warrants caution in interpretation of data, but considering the objective of effecting change into stress perception of the athletes, perhaps this may serve to be useful insight considering Athlete 2’s perceived stress level actually increased rather than decreased. This singular case was actually consistent with the study by Glass et al. (2018) on a similar mindfulness-based intervention on collegiate athletes, whereby higher levels of stress and anxiety were observed, possibly due to increased awareness to anxiety symptoms at least in the short term. Future interventions should consider the function of stress levels (facilitative or non-facilitative) alongside mindfulness-based interventions, if higher stress is to be expected with attainment of better sport performance.

Perhaps the crux of most interventions lie with the final objective: improvements in sports performance. Overall, all athletes experienced improvements in coach-rated performance either during intervention or post-intervention phases, with acute and chronic improvements observed. Consistent with past studies examining mindfulness-based interventions on sport performance (Hasker, 2010; Mistretta et al., 2017; Josefsson et al., 2019; Zadeh et al., 2019), performance in this study was also observed to improve during and after the intervention phase at different rates across individual athletes. Surprisingly, Athlete 1 experienced a minor acute reduction in coach-rated performance during onset of intervention, and even across the intervention phase itself, but rebounded post-intervention to levels above baseline and intervention. This may actually signify that MAC program has been non-facilitative to this particular athletes’ on-the-court performance as observed by the coach. This isolated case was also consistent with the findings in Glass et al. (2018) which found increases in mindful practice not being associated to improved coach-rated performance. Granted, Glass et al.’s (2018) findings were possibly due to the timing of the intervention which occurred during the off-season, hence coaches did not have competition results to compare with, as well as the experience of the participants which were freshmen, hence limited access for observation by coaches. This is contrasted by Athlete 1’s self-rated increases in both acute and chronic performance, where interestingly, performance achieved the peak on the second intervention session. In that vein, coach’s observation may need to be considered alongside some objective measures of sport-specific performance (either technical, physical or tactical) to verify the athlete’s progress.

Findings also show a mixed response to self-rated performance. While four (Athlete 1, 2, 3, and 4) athletes experienced an acute improvement following intervention onset, only three (Athlete 2, 3, and 4) had a sustained effect over the intervention phase. Among them, Athletes 2 and 3 were actually on a decline in self-rated performance during baseline (which reversed to an accelerating trend following the MAC program. Conversely, Athlete 4 who had a stable baseline, experienced a higher decline in performance during intervention, which returned to baseline level upon withdrawal. Further investigation revealed that Athlete 4 had a comparably difficult competition compared to the other athletes, as he/she was consistently matched with higher ranking competitors. Despite positive progress in all other aspects measured and being rated positively by the coach, it simply did not parallel increases in self-rated performance. Future interventions should consider the different adaptations in performance relevant to the individual following mindfulness-based interventions alongside more tangible markers of performance to facilitate athletes’ awareness of their performance, with technical variables such as pass accuracy, runs scored, field goal accuracy (Madden and McGown, 1988) to even anaerobic performance (Khalid et al., 2018).

Athlete 2 presented a peculiar case in the current study, being the only subject to have variability in coach-rated performance level during intervention phase, whereas all other athletes’ levels were determined to be stable throughout. Specifically, Athlete 2’s variability was determined after observing there being 3 data points in intervention phase which were lower than the range of the stability envelope, warranting caution in interpreting improvements in coach rated performance for Athlete 2. Curiously, Athlete 2 also experienced the highest acute increase (upon onset of MAC intervention) in experiential avoidance, and also leading all subjects in chronic changes between baseline and intervention, notably being one of only two to display a large effect size. Despite also attaining the highest amount of chronic MAAS improvements and performance, this signifies an increase, albeit a trivial one, in the subject’s struggle with goal-directed behavior (Gardner and Moore, 2004). A possible explanation is that as the intervention was conducted in the midst of a non-priority competition, the athlete was faced with a dilemma of either focusing on performing or practicing of mindful skills, which may have consequences for goal-directed behavior.

The current study presented a few distinctions, for example, the positive observation of acute and chronic effects due to the MAC program *via* single case research design. Given that much of past studies implemented large scale controlled trials (Lutkenhouse et al., 2007; Hasker, 2010; Gross et al., 2016) with student-athletes and semi-professionals, the current study was conducted real-time in a competitive season for sub-elite athletes, as such changes observed are reflective of the athletes’ conditions sensitive to changes in daily life. The athletes in the current study train and compete year round with no segmentation of seasons, hence observations gained from the current study is gained with the objective of assimilating to real-life situations as compared to lab-based experiments.

The choice of measure for proficiency in mindful practice was also one of contention. Other than being a contemporary measure of trait mindfulness, the MAAS served as the measure for the participants’ proficiency in mindful practice as it was more closely aligned with the study’s objective of observing changes at the weekly level. With trait mindfulness being more trait-like (i.e., stable personality characeristics) in nature, perhaps a pre-post test of personality traits would have served to enrich the findings. Notably, in a meta-analysis examining mindfulness and Big Five personality traits, trait mindfulness has been correlated strongly with neuroticism, negative affect and conscientiousness (Giluk, 2009). Looking at the results, the current study speculates (but not assume) that long term changes in personality traits may be preceded by changes in trait mindfulness, but this needs to be furnished with data. Future studies can consider longitudinal designs observing not only changes at the mindfulness level, but also at the personality level.

The current study should be interpreted with a few limitations to consider. Although trait mindfulness was observed to have increased following the MAC program, the current study did not account for measuring changes in state mindfulness. The degree to which each individual participant experienced states of mindfulness post session was purely speculated and should be controlled for in future studies. Also, the rate at which participants self-organized their mindful practice outside of the group sessions was not controlled for. Although participants were encouraged to self-practice and check in with the researchers, it was not made compulsory and frequency was not recorded. In more controlled settings, future studies should consider controlling for changes in both state mindfulness and participants’ self-practice outside of group sessions. Notably, changes in sport performance were also purely dependent on ratings based on coach’s observations. This reliance may have repercussions in the data due to expectancy and confirmation bias. Future studies should consider pairing this subjective measure with objective measures of performance that may more closely tie-in with on-court performance.

Overall, the current observations lend support to MAC being a viable intervention to improve mindful attention, stress reduction and performance in a sport setting.

### Implications of the Study

The MAC program is well-designed to expound the practice of mindfulness in sport across the athletic population seeking for performance enhancement. The current study investigated the MAC program’s efficacy in a group of sub-elite athletes carrying similar responsibilities to their elite counterparts in the international competitive stage. Based on the results, there are a few major observations that can facilitate prospective practitioners in onboarding such an intervention into their athletic program. Firstly, the 7-week MAC program is capable of effecting short-term benefits to athletes’ mental preparation, particularly proficiency in mindfulness, perceived stress and subsequently sport performance. However, the MAC program should not be taken for granted as a last-minute resort as may often be the case with clients seeking a quick fix in arbitrary situations. MAC effects on performance may take time to manifest, which may extend beyond a practitioner’s expectations (Josefsson et al., 2019). As discussed above, each athlete presents an individualized learning curve, with benefits potentially only surfacing weeks (if not months) later after the conclusion of the 7 weeks program. Due diligence should be paid to observing how each athlete responds to the program, rather than depending solely on sport performance measures. As such, although the MAC program can be slotted into most phases of the annual calendar, it may be wise to treat it as a long-term intervention best suited during general preparatory or even the off-season phases. Future studies can consider longitudinal designs spanning a year or longer to observe for potential long-term benefits.

Secondly, although the MAC program was designed as a linear weekly program which concludes in 7 weeks, practitioners should also consider the fluctuations in physical and psychological stress across a season or even a macrocycle. With sport performance as the object of most athletic programs, contextual and time-specific stress becomes unavoidable as training and competitive stress increases. Practitioners may benefit from monitoring stress levels alongside sport performance, and revisit certain key concepts of the MAC program in anticipation of stressful periods during the season. This may be through 1-day intensive workshops focusing on the mindfulness techniques, individual consultations or through environmental stimulations which encourages practice of mindfulness. Extending the practice of MAC beyond the classroom is one way of encouraging transfer of learning, and can be done through infrastructure (e.g., slogans, banners) or even in team culture by integrating mindfulness practice into coaching practices (e.g., team meditations, skills practice etc.).

Thirdly, while mindfulness approaches may be extensive in theory, practitioners should also consider that sport performance does not occur in a theoretical vacuum. As seen in the current study, individualized responses to the MAC program (or general mindfulness approaches) are to be expected. In a competitive sporting culture that exemplifies meritocracy and outcome-oriented behavior, it may be wise to integrate mindfulness approaches to a compatible overall training concept that fits the athlete rather than insisting on being dogmatic (Birrer et al., 2012). Sport performance remains the object of most training programs, and mindfulness development might be best served as part of the means rather than the end.

## Conclusion

Despite some initial predictions not being supported by the findings, the current study yielded several meaningful insights into the practice of MAC for psychological indices and sports performance which may warrant as a viable method in working with a specific sporting population such as the sub-elite athletes. In a competitive training environment not far removed from elite athletes, having proficiency to practice being present mindfully may help sub-elite athletes achieve a consistent upward progression in performance over a training cycle. The MAC program was observed to concurrently improve athletes’ trait mindfulness and rated sport performance. Improvements in performance was the highest at a delayed time from onset of intervention, signifying a required duration of athletes to internalize mindful skills in order to transfer to sport performance. Contrary to past findings, MAC program may not elicit major changes in experiential avoidance and psychological inflexibility across a short period of 7 weeks, but may require a much longer duration for these traits to have remarkable changes. Perceived stress dropped below baseline for most athletes suggesting an increased ability to self-regulate stress during down-time, however, increased stress may not necessarily be detrimental to sport performance. The current study concludes by lending support to MAC interventions designed to improve sport performance and should be integrated into a well-designed psychological training program.

## Data Availability Statement

The raw data supporting the conclusions of this article will be made available by the authors, without undue reservation.

## Ethics Statement

The studies involving human participants were reviewed and approved by Universiti Malaya Research Ethics Committee UM.TNC2/UMREC – 161. Written informed consent to participate in this study was provided by the participants or their legal guardian/next of kin.

## Author Contributions

PNH conceptualized the idea, collected and analyzed data, and drafted the manuscript. RSKW conceptualized the idea, supervised the research, interpreted the data, and reviewed the manuscript. JPGC supervised the research, interpreted the data, and reviewed the manuscript. All authors contributed to the article and approved the submitted version.

## Conflict of Interest

RSKW was employed by MYWellness and Sport Science Consulting. The remaining authors declare that the research was conducted in the absence of any commercial or financial relationships that could be construed as a potential conflict of interest.

## Publisher’s Note

All claims expressed in this article are solely those of the authors and do not necessarily represent those of their affiliated organizations, or those of the publisher, the editors and the reviewers. Any product that may be evaluated in this article, or claim that may be made by its manufacturer, is not guaranteed or endorsed by the publisher.
